# Development and Characterization of CD44-Targeted X-Aptamers with Enhanced Binding Affinity for Cancer Therapeutics

**DOI:** 10.3390/bioengineering12020113

**Published:** 2025-01-26

**Authors:** Hongyu Wang, Weiguo He, Miguel-Angel Elizondo-Riojas, Xiaobo Zhou, Tae Jin Lee, David G. Gorenstein

**Affiliations:** 1 Department of Diagnostic and Interventional Imaging, McGovern Medical School, University of Texas Health Science Center at Houston, Houston, TX 77030, USA; 2 Institute of Molecular Medicine, McGovern Medical School, University of Texas Health Science Center at Houston, Houston, TX 77030, USA; weighe@gmail.com; 3 Centro Universitario Contra el Cáncer, Hospital Universitario “Dr. José Eleuterio González”, Universidad Autonoma de Nuevo León, Monterrey 64460, NL, Mexico; 4 Center for Computational Systems Medicine, McWilliams School of Biomedical Informatics, University of Texas Health Science Center at Houston, Houston, TX 77030, USA; xiaobo.zhou@uth.tmc.edu; 5 Department of Neurosurgery, McGovern Medical School, University of Texas Health Science Center at Houston, Houston, TX 77030, USA; tae.jin.lee@uth.tmc.edu

**Keywords:** CD44, X-aptamers 2, binding motifs, CD44-expressing cancer cells

## Abstract

CD44, a pivotal cell surface molecule, plays a crucial role in many cellular functions, including cell-cell interactions, adhesion, and migration. It serves as a receptor for hyaluronic acid and is involved in lymphocyte activation, recirculation, homing, and hematopoiesis. Moreover, CD44 is a commonly used cancer stem cell marker associated with tumor progression and metastasis. The development of CD44 aptamers that specifically target CD44 can be utilized to target CD44-positive cells, including cancer stem cells, and for drug delivery. Building on the primary sequences of our previously selected thioaptamers (TAs) and observed variations, we developed a bead-based X-aptamer (XA) library by conjugating drug-like ligands (X) to the 5-positions of certain uridines on a complete monothioate backbone. From this, we selected an XA with high affinity to the CD44 hyaluronic acid binding domain (HABD) from a large combinatorial X-aptamer library modified with N-acetyl-2,3-dehydro-2-deoxyneuraminic acid (ADDA). This XA demonstrated an enhanced binding affinity for the CD44 protein up to 23-fold. The selected CD44 X-aptamers (both amine form and ADDA form) also showed enhanced binding affinity to CD44-overexpressing human ovarian cancer IGROV cells. Secondary structure predictions of CD44 using MFold identified several binding motifs and smaller constructs of various stem-loop regions. Among our identified binding motifs, X-aptamer motif 3 and motif 5 showed enhanced binding affinity to CD44-overexpressing human ovarian cancer IGROV cells with ADDA form, compared to the binding affinities with amine form and scrambled sequence. The effect of ADDA as a binding affinity enhancer was not uniform within the aptamer, highlighting the importance of optimal ligand positioning. The incorporation of ADDA not only broadened the XA’s chemical diversity but also increased the binding surface area, offering enhanced specificity. Therefore, the strategic use of site-directed modifications allows for fine-tuning aptamer properties and offers a flexible, generalizable framework for developing high-performance aptamers that target a wide range of molecules.

## 1. Introduction

CD44 is a transmembrane glycoprotein consisting of an extracellular domain, a transmembrane domain, and a cytoplasmic tail. The extracellular domain contains binding sites for hyaluronic acid and other ligands. Although various splicing variants of CD44 are expressed on the cell membrane of cancer cells, the hyaluronic acid binding domain (HABD) is highly conserved among the CD44 splicing variants. Its role as a receptor for hyaluronic acid (HA) [1] makes it a key player in cell-cell interactions, adhesion, and migration. Through alternative splicing, the CD44 gene produces two main isoforms: the standard CD44s isoform and the variant CD44v isoforms. CD44v has a specific binding site for HA on its extracellular domain, allowing for a strong interaction between the two molecules. The interaction of CD44v with HA is considered a key factor in tumor growth and metastasis [2]. CD44 is overexpressed in many cancer cells and cancer stem cells. Its overexpression has been linked to poor prognosis and aggressive biological behaviors in many cancer types [3,4]. Therefore, CD44 is a commonly used cancer stem cell (CSC) marker associated with tumor progression and metastasis and promotes epithelial-mesenchymal transition [5,6,7]. The capabilities of CSCs for self-renewal and differentiation contribute to tumor recurrence and resistance to therapy.

Consequently, CD44 is a promising therapeutic target for various cancers, including prostate, colon, breast, ovarian, and pancreatic cancers [8]. Targeting strategies include the development of antibodies, small molecules, and hyaluronic acid-based therapies to inhibit CD44 function [9]. Specifically, HA-CD44 interactions can be exploited to deliver chemotherapeutic drugs and other anticancer agents to cancer cells. Many investigators have shown increased efficacy in cell and animal tumor models by conjugating drugs to HA or anti-CD44 antibodies and incorporating drugs or siRNAs into vehicles decorated with HA or antibodies [10,11].

Our study focuses on developing specific aptamers that can bind to the HABD of CD44 with high affinity and specificity. Aptamers are single-stranded oligonucleotides that can bind to specific targets with high affinity and specificity due to their unique three-dimensional structures. They are attractive molecular tools for targeting proteins like CD44 due to their versatility, low immunogenicity, and ease of chemical modification, and they have shown considerable promise in therapeutic applications [12]. Successful clinical trials have demonstrated aptamer safety and efficacy [13,14], leading to ongoing research focusing on optimizing selection and stability, enhancing specificity, and expanding applications in personalized medicine and targeted therapy. 

Thioaptamers (TAs) are a subtype of aptamers that contain sulfur modifications to enhance their binding affinity and stability [15]. Over the past years, we developed libraries of nucleic acids with phosphorothioate modifications, allowing for the selection of thioaptamers with enhanced affinity and specificity, such as transcription factor NF-κB [16], AP-1 proteins regulating cytokine expressions during virus infection [17], CD44 HABD [15], E-selectin protein [18], vimentin [19], and annexin A2 [20]. However, there is a continuous need for further optimization to improve their therapeutic potential. This includes improving their stability, binding affinity, and specificity to ensure they can effectively target and modulate biological processes. Moreover, conventional aptamer selection methods, such as SELEX, are often time-intensive and require multiple rounds of iterative selection and amplification. To address these challenges, X-aptamer technology has been developed. An X-aptamer is a modified aptamer with “drug-like” molecules (X) randomly attached to specific uridine bases in the DNA backbone (5-X-dU bases), allowing for seamless sequencing via Taq polymerase [21,22]. These added molecules improve the aptamer’s binding affinity and specificity by improving interaction interfaces, such as hydrophobic contacts and structural stabilization, particularly in the hairpin loops [23,24]. The X-ligands can range from amino-acid-like side chains to a complex drug moiety, providing unlimited functional possibilities beyond the standard four DNA bases or 20 amino acids. We create a highly diverse and complex library of X-aptamers by combining the beads-based thioaptamer selection method [25,26] with these additional X-ligands. This approach also simplifies the selection process, requiring only one round and two steps [27], making it efficient and versatile for various applications.

In this study, we report the development and characterization of CD44-targeted X-aptamers using a beads-based library approach. This method integrates functionalized beads with pre-modified libraries to rapidly and efficiently select high-performance aptamers. Among the modifications explored, the ADDA (azido-dibenzylamine) group was identified as a critical enhancer of binding affinity, outperforming traditional amino (NH_2_) modifications. Importantly, the binding efficiency of ADDA-modified aptamers was shown to depend on their modification position within the sequence and the structural context, particularly in the hairpin loop regions. By leveraging these advancements, this work demonstrates a streamlined, high-throughput platform for selecting X-aptamers with optimized chemical diversity and enhanced functional performance against CD44, offering potential applications in targeted cancer diagnostics and therapeutics.

## 2. Materials and Methods

### 2.1. Materials

The dA, dG, dC, and dT cyanoethylphosphoramidites and the Beaucage reagent (3H-1,2-benzodithiol-3-one 1,1-dioxide) were purchased from Glen Research (Sterling, VA, USA). The *Taq* polymerase kits were from Applied Biosystems (Foster City, CA, USA). Polystyrene beads (60–70 µm) with non-cleavable hexaethyleneglycol linkers loading 36 µmol/g were from ChemGenes Corp. (Ashland, MA, USA). The oligodeoxynucleotides (ODNs) and monothioated S-ODNs used in the study were synthesized on a 1 µmol scale in an Expedite 8909 System (Applied Biosystems, Foster City, CA, USA) DNA synthesizer. NHS-PEG12-biotin was purchased from Pierce (Thermo Fisher Scientific, Rockford, IL, USA). *N*-acetyl-2,3-dehydro-2-deoxyneuraminic acid (ADDA) was purchased from Sigma-Aldrich (St. Louis, MO, USA). CD44-HABD protein was produced in our laboratory by cloning the DNA sequences encoding CD44-HABD (20–178 amino acid residues) into expression vector pET19b between the NdeI and BamHI sites and following published procedures [28] for protein expression, refolding, and purification.

### 2.2. Synthesis of Monothioated Oligodeoxynucleotides (S-ODN) Library

Based on the primary sequence of our selected thioaptamers that substituted with monothiophosphates on the 5′ side of dA and bound to the hyaluronic acid binding domain of CD44 (CD44-HABD) [15], we synthesized a pseudorandom, beads library of aptamers using an automated four-column, split-pool synthesizer [29]. This synthesis produced a library with >1 million (4^10^) unique thioaptamer sequences. Using DOCK6.4 software [30] on the nine representative protein structures with the 2798 small molecules, the ADDA ligand (N-acetyl-2,3-dehydro-2-deoxyneuraminic acid, PubChem: CID 65309) was selected as lead compound [21]. After synthesis, ADDA was conjugated to certain 5-(aminoethyl-3-acrylimido)-deoxyuridine (amino-dU) of the original XA library using amine-carboxy coupling [21]. The beads-based library consists of a 5′-primer region (5′-GAGATTCATCACGCGCATAGTC-3′), a 30 nucleotide pseudo-random sequence, and a 3′-primer region (5′-CGACTATGCGATGATGTCTTC-3′) that is covalently linked to a 65-µm polystyrene bead (ChemGenes) by a non-cleavable hexaethyleneglycol linker.

### 2.3. CD44-HABD Specific X-Aptamer Selection

First, we carried out a negative selection by incubating the synthesized X-aptamer beads library with streptavidin-coated magnetic Dynabeads (Thermo Fisher Scientific, Waltham, MA, USA) and collected the non-binding X-aptamers for positive selection. After labeling the CD44-HABD protein with NHS-PEG12-biotin (Thermo Fisher Scientific), the biotinylated CD44-HABD was first incubated with the non-binding X-aptamers from the negative selection, followed by incubation with streptavidin-coated magnetic Dynabeads (Thermo Scientific). The binding complex of X-aptamer with biotinylated CD44-HABD and streptavidin-coated magnetic Dynabeads was selected using a magnetic stand. After selection, the CD44-HABD specific X-aptamers were cleaved using 50 µL 1N NaOH at 65 °C for 30 min, followed by neutralization with 40 µL 2M Tris-Cl. The cleaved putative aptamers were PCR amplified and sequenced by next-generation sequencing (NGS). The sequence of the X-aptamer was determined by the sequencing analysis, and the original library design was referenced to determine the X positions [21].

### 2.4. Filter Binding Assay

The equilibrium binding constants of selected XAs for CD44-HABD were determined by a filter binding assay. The biotinylated XAs (1 nM) were incubated with varying concentrations of CD44-HABD in 50 µL of 20 mM Tris buffer (150 mM NaCl, pH 8.0) for 40 min at room temperature and then transferred to a 96-well dot-blot apparatus and filtered under vacuum onto nitrocellulose membranes, which retain the CD44-HABD along with any bound XAs. After washing, the amount of biotinylated XA retained at each spot was determined by chemiluminescent detection using the Chemiluminescent Nucleic Acid Detection Module (Thermo Scientific) following the manufacturer’s instructions. The chemiluminescent signals were collected on a Chemimager (Alpha Innotech, San Leandro, CA, USA). Image analysis and quantification of spot intensities were performed using Image J, Version 1.54 [31]. Binding analysis was based on the spot intensities on the nitrocellulose membranes with subtraction of background spot intensity due to the buffer effect from all the data points. Saturation binding curves were generated by using GraphPad Prism with curve fits. The equilibrium dissociation constants KD were calculated from the equation Y = Bmax X/(Kd + X), assuming a single binding site.

### 2.5. Cell Lines and Cell Binding Assay

The human ovarian carcinoma cell line IGROV-1 cells were a kind gift from A.K. Sood (University of Texas M.D. Anderson Cancer Center, Houston, TX, USA). The IGROV-1 cells were maintained in RPMI-1640 medium supplemented with 15% fetal bovine serum and 0.1% gentamicin sulfate. The CD44 negative NIH3T3 fibroblast cell line (ATCC, Manassas, VA, USA) was maintained in Dulbecco’s modified Eagle’s medium (DMEM) supplemented with 10% fetal bovine serum. All experiments were performed with 70–80% confluent cultures with 5% CO_2_ at 37 °C. After screening for CD44 expression using an anti-human CD44 antibody (BioLegend, San Diego, CA, USA), IGROV-1 was used as CCD44-positive cells, and NIH3T3 was used as CD44-negative control. For the immunofluorescence microscopy assay, IGROV-1 cells were seeded in chamber slides (Cole-Parmer, Vernon Hills, IL, USA) and incubated with the biotinylated XAs at different concentrations after blocking with a universal blocking buffer (Thermo Fisher Scientific, Waltham, MA, USA). The binding of the XA motifs to IGROV-1 cells was measured by fluorescence intensity after incubating with CD44-fluorescein isothiocyanate (FITC) or XA-Cy3.

### 2.6. Flow Cytometry Analysis of Aptamer Binding

IGROV-1 and NIH3T3 cells were blocked with a universal blocking buffer before incubation with biotinylated XAs, biotinylated motifs, or thioaptamer controls (TA1 and TA2) at room temperature for 1 h. After washing to remove excess XAs, fluorescein-labeled streptavidin (BioLegend, San Diego, CA, USA) was incubated with cells for 30 min at room temperature. The binding of XAs or motifs to CD44 expression IGROV-1 cells or NIH3T3 cells was measured by the percentage of positive cells and fluorescence intensity with FACScalibur flow cytometry (BD Biosciences, San Jose, CA, USA). Anti-human CD44 antibody (BioLegend, San Diego, CA, USA) was used as a positive control.

## 3. Results

### 3.1. Selection of High-Affinity CD44 X-Aptamers

**Figure 1:**
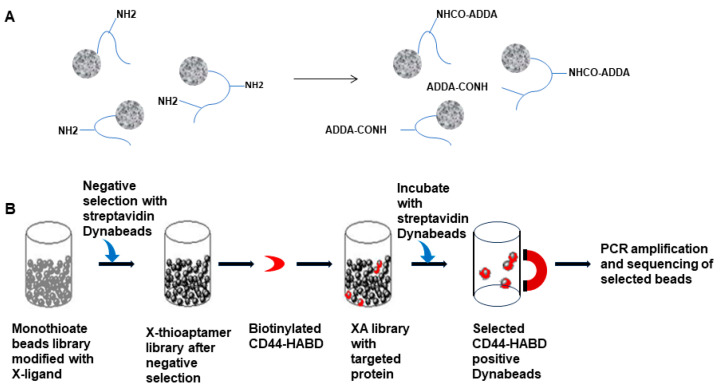
Modification and selection of CD44-HABD-specific X-aptamers. (**A**). Illustration of conjugate ADDA to the XA sequences by amine-carboxy coupling method. (**B**). Steps of selecting CD44-HABD specific X-aptamers using beads-based X-aptamer library.

We synthesized a pseudorandom one-bead-one-sequence library containing over 1 million (4^10^) unique X-aptamer sequences. ADDA, which was selected based on the interactions with CD44-HABD protein [21], was conjugated to the amino-dU of these sequences using carbodiimide chemistry (Figure 1A). This modified bead-based X-aptamer library was employed for a selection process to identify X-aptamers (XAs) with specificity to CD44-HABD (Figure 1B). Both amine- and ADDA-modified XAs were evaluated in this study. The top CD44-HABD-specific XA sequences identified are shown in Table 1.

**Figure 2:**
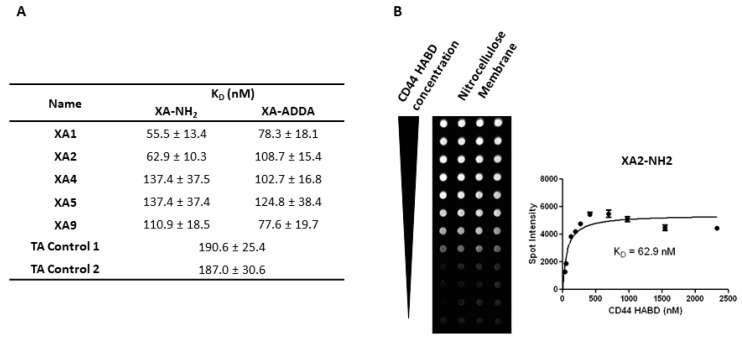
Equilibrium dissociation constants of selected monothioated XAs binding to CD44-HABD protein. (**A**) The equilibrium dissociation constants (Kd) of different XAs modified with ADDA or NH2. (**B**) Representative quadruplicate spot image and the saturation binding curve for the biotinylated XA2-NH2 protein. Chemiluminescent detection of spot intensities on nitrocellulose membranes was used to quantify the binding affinity of XAs.

To further characterize these XAs, we determined their equilibrium binding constants to the CD44-HABD protein using a filter binding assay. Biotinylated XAs (1 nM) were incubated with varying concentrations of CD44-HABD protein, and the amount of bound biotinylated XAs retained at each spot was quantified. From these data, equilibrium dissociation constants (KD) were calculated and are summarized in Figure 2A. Lower KD values signify higher binding affinity. Among the XAs tested, XA1 (KD = 55.5 ± 13.4 nM for XA-NH_2_) and XA2 (KD = 62.9 ± 10.3 nM for XA-NH_2_) showed the highest binding affinity in the XA-NH_2_ group. The ADDA-modified versions generally displayed higher KD values, suggesting slightly reduced binding affinity relative to their XA-NH_2_ counterparts. Compared to the control aptamers (KD = 190.6 ± 25.4 nM for TA1 and KD = 187.0 ± 30.6 nM for TA2), both XA-NH2 and XA-ADDA groups demonstrated significantly lower KD values, indicating the improved binding affinity for CD44-HABD with the modifications of NH2 or ADDA. Figure 2B displays the spot intensity of XA2-NH2 binding to CD44-HABD protein on a nitrocellulose membrane, demonstrating the binding strength across different protein concentrations. The binding curve of represented XA2-NH2 indicates a KD value of 62.9 nM, confirming its high affinity to CD44-HABD protein. This data supports the potential of XA2-NH2 and other high-affinity XAs as effective molecular probes for targeting CD44-HABD.

### 3.2. Identification of Binding Motifs

**Figure 3:**
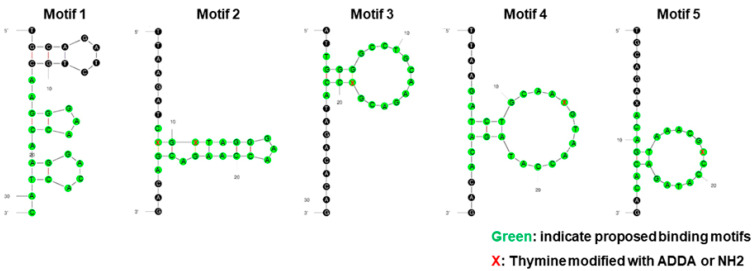
Secondary structures of XAs predicted using MFold. Proposed binding motifs are shown in green.

The secondary structures of selected X-aptamers (XAs) were predicted using MFold, Version 1.54, highlighting structural elements such as hairpin loops and stem regions (Figure 3). The predicted secondary structures for XA1, XA2, XA4, XA5, and XA9 reveal the potential for distinct hairpin loop formations, with specific regions of the sequences highlighted in green to indicate proposed secondary structures and binding motifs. The principle of our truncation is based on the stem and loop structures of the predicted secondary structures. These motifs, located within the random region, are hypothesized to contribute to the binding specificity and affinity of the XAs to CD44-HABD.

To investigate the structural elements responsible for target interaction, confirming that the green-highlighted regions are likely critical for the high-affinity binding observed in these X-aptamers, five motifs representing various X-aptamer stem-loop regions were synthesized and analyzed for binding efficacy. We tested their binding affinity to CD44-HABD protein using a filter binding assay and demonstrated improved binding affinity of all five motifs compared to their original XAs based on their KD values (Table 2).

### 3.3. Binding Affinity to CD44-Expressing IGROV Cells

**Figure 4:**
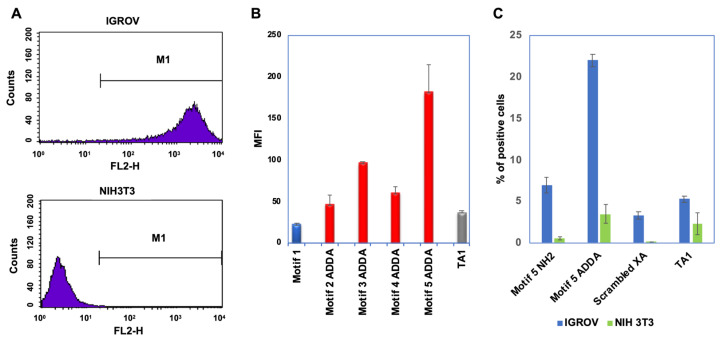
Cell binding affinity and specificity of the motifs’ amine form and ADDA form. (**A**). CD44 overexpression human IGROV cells and CD44 low expression NIH3T3 fibroblast cells served as positive and negative cells, respectively, in this study. (**B**). Mean fluorescence intensities (MFI) of Cy3-labeled motifs binding to IGROV cells. IGROV cells were incubated with selected motifs at room temperature for 30 min. A non-modified thioaptamer (TA1) was used as a negative control. Motifs 3 and 5 exhibited strong binding to IGROV cells, while motif 1 had no modification. (**C**) This demonstrated the specificity of motif 5 binding to CD44-expressing IGROV cells, compared to CD44 negative NIH3T3 cells, as well as the non-modified TA control.

To validate the binding affinity of the selected binding motifs with live cells, we first examined the cell lines with or without CD44 expression for the study. As demonstrated in Figure 4A, human IGROV cells overexpress CD44 on the cell surface, while NIH3T3 fibroblast cells expressed a low level of CD44 antigen. Therefore, we used IGROV cells for our downstream binding affinity examination and NIH3T3 cells as negative control. To assess the performance of the selected CD44-ADDA binding motifs, both the amine (NH_2_) and ADDA-modified forms were tested, with the ADDA-modified motifs outperforming their NH_2_ counterparts. The non-modified thioaptamer (TA1) served as a negative control.

We used multiple ovarian cancer cell lines to validate the binding of the selected XAs and motifs, including CD44-positive IGROV and SKOV3 cell lines, as well as the CD44-negative A2780 cell line. Both IGROV and SKOV3 cells demonstrated similar binding performance to the selected XAs and motifs. We selected IGROV cells as representative CD44-positive cells for this manuscript to streamline the presentation of our findings. Among the identified binding motifs, motif 3 and motif 5 exhibited the strongest binding to CD44-overexpressing IGROV cells. Cy3-labeled motif 5 showed strong binding to IGROV cells, with no binding observed in control experiments using TA1 or scrambled XA (Figure 4B). Specificity studies confirmed that these motifs are selectively bound to CD44-expressing IGROV cells, with no detectable binding to NIH 3T3 cells, which lack CD44 expression (Figure 4C). This specificity underscores the potential of these X-aptamers as targeted molecular tools for CD44-positive cells.

### 3.4. Modification of ADDA Outperforms NH2 in Enhancing Binding Affinity

**Figure 5:**
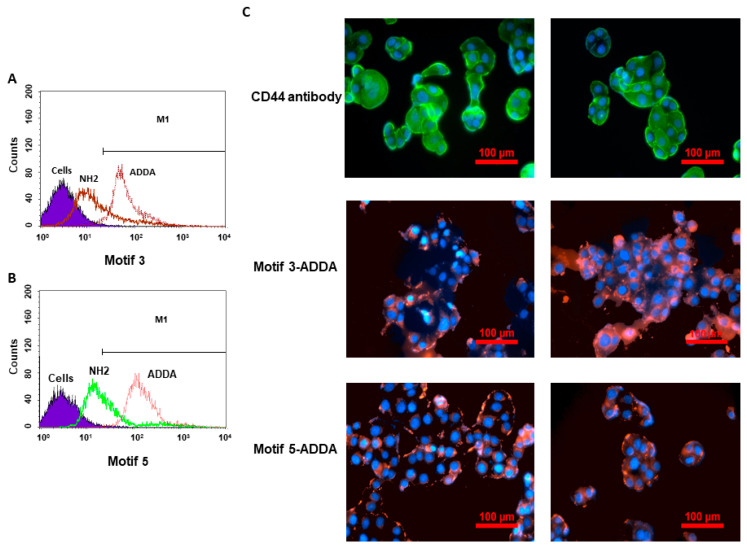
Analysis of CD44 binding by specific motifs using flow cytometry and fluorescence microscopy. (**A**,**B**) Flow cytometry analysis of cells treated with NH2- and ADDA-modified motif 3 and motif 5, respectively. Fluorescence intensity histograms indicate binding efficiency, with ADDA-conjugated motif 3 or motif 5 showing a higher fluorescence shift than NH2. (**C**) Fluorescence microscopy images of cells stained for CD44 were analyzed with motif 3 and motif 5 in ADDA form. The top row shows CD44 localization with a green fluorescent antibody, confirming CD44 expression on the cell surface. The middle row shows the binding of X-CD44 Cy3-conjugated motif 3 (red), with blue nuclei counterstained with DAPI. The bottom row displays X-CD44 Cy3-conjugated motif 5 binding (red) with corresponding nuclear staining (blue). Scale bars = 100 µm.

We conducted experiments to study the binding affinity of both the amine form and ADDA form modifications. Using flow cytometry and fluorescence microscopy, we compared the fluorescence intensity of motif 3 and motif 5 with IGROV cells. A CD44 antibody was used as a positive control. Figure 5A,B prospectively demonstrated the shifts in the fluorescence peaks of motif 3 and motif 5, indicating specific binding of NH2 and ADDA-modified motifs to CD44 on the cell surface. ADDA form produced a higher fluorescence intensity than NH2 in both motif 3 and motif 5, indicating stronger binding to CD44 on the IGROV cells. Therefore, the flow cytometry data (A, B) suggest differences in binding efficiency or specificity between NH2 and ADDA conjugates for motifs 3 and 5. Figure 5C represents images visualizing the expression and localization of CD44 protein on IGROV cells using fluorescent antibodies and Cy3-conjugated motif 3 and motif 5 in ADDA form. CD44 antibody staining (top row) confirms the presence of CD44, mainly localized on the cell surface. Cy3-labeled motifs (middle and bottom rows) demonstrate specific binding to CD44, with motif 5 showing stronger staining intensity than that with motif 3. Therefore, the microscopy data (C) validate the localization of CD44 on the cell surface and the specific binding of the motifs, as seen in the red fluorescence.

## 4. Discussion

### 4.1. Comparing the Current Method with Existing Antibody and Aptamer Technologies

The current method of X-aptamers offers several advantages over traditional antibody and aptamer technologies, particularly in targeting CD44-positive cells, including cancer stem cells. By conjugating drug-like ligands to the aptamer backbone, this method enhances binding affinity and chemical diversity, with up to 23-fold improvement in affinity for the CD44 hyaluronic acid binding domain (HABD). X-aptamers are synthetically produced, offering better stability and scalability compared to antibodies, which are prone to production costs, immunogenicity, and stability issues. However, challenges remain, such as optimizing ligand positioning for consistent performance and achieving binding affinity comparable to antibodies for all targets. Despite these challenges, the current method holds significant potential to overcome the limitations of both antibodies and traditional aptamers in molecular recognition applications. The development of CD44-targeted X-aptamers represents a significant advancement in aptamer selection methodologies, driven by achieving high binding affinity and selectivity with efficiency in both time and procedural complexity. CD44, a cell surface glycoprotein, is implicated in various physiological and pathological processes, including cancer progression and metastasis, making it an attractive therapeutic and diagnostic target. Our findings highlight X-aptamers’ potential in targeting CD44-positive cancer cells, including cancer stem cells, which are often resistant to conventional therapies.

### 4.2. Beads-Based X-Aptamer Library: Streamlining Selection and Enhancing Affinity

The beads-based X-aptamer library is an innovative platform that significantly simplifies the traditional aptamer selection process. Conventional aptamer selection methods, such as SELEX (Systematic Evolution of Ligands by Exponential Enrichment), often involve multiple rounds of selection, amplification, and characterization, requiring considerable time and effort. The beads-based X-aptamer library eliminates many of these steps by incorporating chemical modifications directly to the uridine bases in the DNA backbone (5-X-dU bases), allowing for seamless sequencing via Taq polymerase. These modifications can enhance binding affinity and specificity by introducing functional groups that strengthen interactions with the target and stabilize the aptamer structure, particularly in critical regions like hairpin loops. It also reduces off-target interactions and improves the functional performance of the aptamers, making them more reliable for therapeutic and diagnostic applications. Moreover, integrating these modifications during the aptamer selection process, as opposed to post-selection, saves time and reduces development complexity. In this work, the incorporation of ADDA exemplifies how these modifications enhance binding by broadening the interaction surface and optimizing spatial alignment with the target.

Our bead-based library design and selection process can be adapted to a wide range of molecular targets, making it a versatile tool for developing high-affinity aptamers. By incorporating diverse chemical modifications (ranging from amino-acid-like side chains to a complex drug moiety) during the selection phase, this approach enables efficient identification of aptamers with enhanced binding properties. Moreover, our methodology extends beyond the use of a single modification type. For instance, multiple drug hits can be incorporated into a single aptamer. This allows for the creation of aptamers with enhanced combinations of binding moieties, broadening their functional capabilities. Therefore, the strategic use of site-directed modifications allows for fine-tuning aptamer properties and offers a flexible, generalizable framework for developing high-performance aptamers targeting a wide array of molecules.

### 4.3. Binding Differences of X-Aptamers with Pure CD44-HABD and CD44-Expressing IGROV Cells

The binding performance of CD44-targeted X-aptamers exhibited distinct differences when tested against purified CD44-HABD and CD44-expressing IGROV cells. These differences are likely due to variations in the molecular environment and the presentation of the CD44 protein in these two contexts. In experiments with purified CD44-HABD, the binding affinity of X-aptamers was primarily influenced by direct molecular interactions between the aptamer and the target. The high-affinity binding observed with purified CD44-HABD highlights the aptamers’ capacity to recognize and interact with the Hyaluronan Binding Domain, a key functional region of CD44. The presence of chemical modifications, such as ADDA, further enhanced the binding by stabilizing the interactions through hydrophobic contacts and π-π stacking, or hydrophobic contacts that are less pronounced with NH2 modifications, which primarily contribute through polar or ionic interactions, particularly in aptamers with modifications positioned in critical structural regions like the hairpin loops. However, when tested on IGROV cells, which naturally express CD44 on their surface, the binding profile of X-aptamers showed variability. Although the aptamers maintained their ability to bind CD44, their binding affinities were influenced by several factors unique to the cellular context, including dynamic and heterogeneous environment, different interactions between aptamer structure and complex cell surface, and steric hindrance from other surface proteins. Additionally, aptamers with ADDA modifications may have differential binding performance depending on the orientation of CD44 and the local microenvironment on the cell surface. Moreover, as our selected aptamers specifically target the CD44-HABD region, whereas the commercially available CD44 antibodies do not exclusively bind to this domain, direct co-localization using these antibodies may not yield conclusive evidence regarding HABD-specific binding.

The discrepancy in binding between purified CD44-HABD and IGROV cells underscores the importance of testing aptamers in both simplified and biologically relevant systems. While purified protein assays are valuable for identifying high-affinity binders, cellular assays reveal functional performance in more realistic conditions, accounting for the complexity of protein expression and localization. The observed binding differences suggest that future optimization of X-aptamers should include considerations of target presentation and cell-surface complexity to ensure robust performance in therapeutic and diagnostic applications.

### 4.4. Influence of ADDA Modification on Binding Affinity: Position and Structural Considerations

The incorporation of ADDA as a ligand enhanced the binding affinity of the X-aptamers. ADDA broadens the XA’s chemical diversity and increases the binding surface area, offering enhanced specificity. However, this effect was not uniform across all the modified aptamers. Its effectiveness highly depends on its position within the aptamer sequence and the structural context, particularly the hairpin loop structure. As aptamers are single-stranded nucleic acids that fold into unique three-dimensional structures, the positioning of functional modifications and the spatial relationship between the aptamer and its target can profoundly influence their binding capabilities [32,33].

Hairpin loops play a critical role in target recognition, serving as the primary binding interface. Incorporating ADDA modifications in these regions can significantly enhance interactions with CD44 by stabilizing the hairpin structure and promoting optimal spatial alignment with the target. Conversely, modifications placed outside critical binding regions or in positions that disrupt the aptamer’s folding may reduce binding affinity. This may explain why certain modification positions negatively impacted targeted binding in our study. Further investigation is needed to examine factors such as the impact of modification positions within the binding region, alterations in structural conformations, and changes in electrostatic interactions that specific modifications might induce.

Structural analyses of ADDA-modified aptamers have revealed that the loop structure’s integrity and the precise positioning of the ADDA group are crucial for maximizing binding efficacy. This finding emphasizes the need for careful design and characterization of aptamers to ensure that chemical modifications enhance, rather than hinder, their functional properties.

## 5. Conclusions

Developing CD44-targeted X-aptamers via a beads-based library represents a significant step forward in aptamer technology. The ability to integrate chemical modifications during the selection process has streamlined development and produced aptamers with improved binding affinities. Among these, ADDA modifications stand out for their ability to enhance binding affinity, provided they are strategically positioned within the aptamer’s structure. These advancements hold promise for the rapid and efficient creation of high-performance aptamers for use in diagnostics, therapeutics, and other biomedical applications. Future work could focus on refining the structural characterization of modified aptamers and exploring their functional performance in vivo.

## Figures and Tables

**Table 1:** Identified top CD44-HABD-specific XA sequences.

Name	Sequences of XAs
XA1	TTAA-GATC-XGX-TAG-GGA-ACC-AAG-ACG-AC-AG
XA2	TGCA-GATC-TGC-AAG-GGA-ACC-AAG-GAC-AC-TAC
XA4	TTGG-GGCC-TGC-AAG-ACG-XCC-ATA-GAC-AC-AG
XA5	TGCA-GAXA-CAG-TAA-ACG-XCC-ATA-GAC-AC-AG
XA9	TTAA-GATC-TGC-AAX-GTA-ACC-ATA-GAC-AC-AG

X: Thymine modified with ADDA or NH2.

**Table 2:** Comparing the equilibrium dissociation constant between the selected motifs and their original XAs.

Name	XA K_D_ (nM)		Motif K_D_ (nM)	
	NH_2_ XA	ADDA Modified XA	NH_2_ Motif	ADDA Modified Motif
XA1/Motif 2	55.5 ± 13.4	78.3 ± 18.1	48.0 ± 18.0	15.5 ± 3.2
XA2/Motif 1	62.9 ± 10.3	108.7 ± 15.4	15.0 ± 2.0	
XA4/Motif 3	137.4 ± 37.5	102.7 ± 16.8	81.2 ± 30.9	64.8 ± 13.7
XA5/Motif 5	137.4 ± 37.4	124.8 ± 38.4	18.0 ± 3.7	10.1 ± 2.6
XA9/Motif 4	110.9 ± 18.5	77.6 ± 19.7	35.4 ± 7.4	13.6 ± 3.0

Motif 1 sequence does not contain any X modification.

## Data Availability

All data generated or analyzed during this study are included in this published article.
